# Defining an Adequate Sample of Earlywood Vessels for Retrospective Injury Detection in Diffuse-Porous Species

**DOI:** 10.1371/journal.pone.0038824

**Published:** 2012-06-26

**Authors:** Estelle Arbellay, Christophe Corona, Markus Stoffel, Patrick Fonti, Armelle Decaulne

**Affiliations:** 1 Laboratory of Dendrogeomorphology, Institute of Geological Sciences, University of Berne, Berne, Switzerland; 2 Institute for Environmental Sciences, University of Geneva, Carouge-Geneva, Switzerland; 3 Landscape Dynamics, Swiss Federal Research Institute WSL, Birmensdorf, Switzerland; 4 CNRS Geolab, University Blaise Pascal, Clermont-Ferrand, France; DOE Pacific Northwest National Laboratory, United States of America

## Abstract

Vessels of broad-leaved trees have been analyzed to study how trees deal with various environmental factors. Cambial injury, in particular, has been reported to induce the formation of narrower conduits. Yet, little or no effort has been devoted to the elaboration of vessel sampling strategies for retrospective injury detection based on vessel lumen size reduction. To fill this methodological gap, four wounded individuals each of grey alder (*Alnus incana* (L.) Moench) and downy birch (*Betula pubescens* Ehrh.) were harvested in an avalanche path. Earlywood vessel lumina were measured and compared for each tree between the injury ring built during the growing season following wounding and the control ring laid down the previous year. Measurements were performed along a 10 mm wide radial strip, located directly next to the injury. Specifically, this study aimed at (i) investigating the intra-annual duration and local extension of vessel narrowing close to the wound margin and (ii) identifying an adequate sample of earlywood vessels (number and intra-ring location of cells) attesting to cambial injury. Based on the results of this study, we recommend analyzing at least 30 vessels in each ring. Within the 10 mm wide segment of the injury ring, wound-induced reduction in vessel lumen size did not fade with increasing radial and tangential distances, but we nevertheless advise favoring early earlywood vessels located closest to the injury. These findings, derived from two species widespread across subarctic, mountainous, and temperate regions, will assist retrospective injury detection in *Alnus*, *Betula*, and other diffuse-porous species as well as future related research on hydraulic implications after wounding.

## Introduction

Tree-ring studies have only recently started to take advantage of the development of automatic image analysis systems, which have enabled extensive examination of xylem cells across series of annual rings with the aim of better comprehending the effects of external factors on tree growth [1]–[2]. As for broad-leaved trees, earlywood vessels of ring-porous species have received particular attention due to their easy recognition and thus facilitated measurement, and have repeatedly shown to contain valuable environmental signals that could be extracted and interpreted retrospectively [3]–[7]. However, García-González and Fonti [8] highlighted the fact that there was no common practice in the sampling procedure of vessels in dendroecological studies and that an adequate sample of cells ought to be established to minimize the risk of missing environmental information. The authors tackled this methodological issue for two ring-porous species, *Castanea sativa* Mill. and *Quercus petraea* Liebl., by researching a representative sample of earlywood vessels that would encode the same climatic signal.

By contrast, not only have there been much fewer dendroecological studies conducted with vessels of diffuse-porous species [9]–[14], but the question of ensuring a representative sample of cells has not been addressed yet. The underlying reasons may revolve around their much higher total number of vessels in each ring, where conduits gradually get narrower with increasing radial distance and only get sparser towards the end of the ring [15]. As a result, previous measurements of vessels in diffuse-porous wood have been made either on a fixed number of randomly selected cells [11], within a specific portion of the ring (early earlywood [16] or central portion of the ring [17]) or across the entire ring [10], [14].

The present study was designed in the context of cambial injury and subsequent formation of narrower vessels. Wounded individuals of grey alder (*Alnus incana* (L.) Moench) and downy birch (*Betula pubescens* Ehrh.) were analyzed to (i) determine the intra-annual duration and local extension of vessel narrowing close to the wound margin and (ii) define an adequate sample of earlywood vessels (number and intra-ring location of cells) for retrospective injury detection based on vessel lumen size reduction. Dating injuries with vessel anomalies is methodologically relevant when associated with increment coring. It should then allow the accurate dating of visible injuries through minimally destructive sampling and, although usually thought compromised via this sampling technique, the possible dating of internally hidden injuries.

## Methods

### Wood Material and Preparation of Microsections

**Figure 1:**
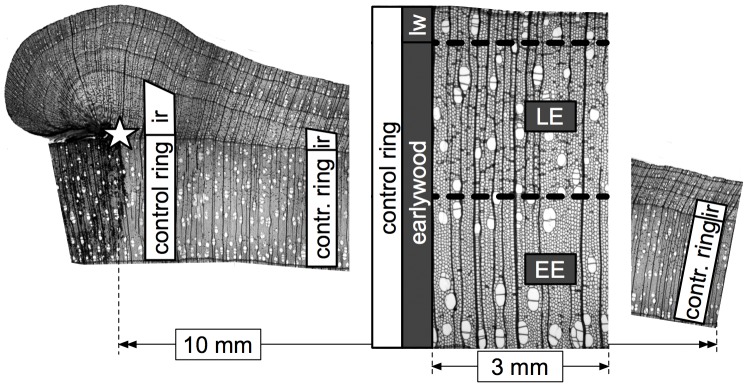
Cambial injury and analysis of earlywood vessels. Cambial injury occurred during the winter of 2006–2007 and trees were harvested in summer 2010. Earlywood vessels were measured in the injury ring (ir) built during the growing season following wounding and in the control ring laid down the previous year. Tree rings in diffuse-porous species consist almost entirely of earlywood, subdivided here in early earlywood (EE) and late earlywood (LE), whereas latewood (lw) is confined to a very narrow terminal zone mostly made of ground tissue. In both rings, measurements were performed over the whole earlywood within a tangential distance of 10 mm from the wound margin (star).

In summer 2010, four wounded individuals each of grey alder (*Alnus incana* (L.) Moench) and downy birch (*Betula pubescens* Ehrh.) were harvested in an avalanche path situated at about 700 m a.s.l. in the Bødalen valley (inner Nordfjord, western Norway, 61°48′ N/7°05′ E). Trees were scarred on the stem by a snow avalanche that occurred during the winter of 2006–2007 (Figure 1). One cross-section per tree was taken at the mid-length of the main injury. Average tree age (ATA) was 29.50 yr (±12.93) and average stem circumference at breast height (ASC) was 21.36 cm (±8.42). Samples (eight cross-sections in total) were sectioned with a chisel to obtain a small wood block (2×1.5×1.5 cm) of the xylem tissue located directly next to the injury. After dating the rings, 15 µm thick transverse sections of the wood blocks were cut using a Reichert sliding microtome. The microsections were then stained with a 1% safranin and astrablue solution, rinsed with water, alcohols and xylol, and permanently mounted on microscope slides using Canada balsam.

### Wood Anatomical Analysis

Earlywood vessels were studied in two successive rings for each tree (16 rings in total): the injury ring built during the growing season following wounding and as a control the ring laid down the previous year (Figure 1). In both rings, vessels were analyzed over the whole earlywood within a tangential distance (or extent around the tree circumference) of 10 mm from the wound margin. Tree rings in diffuse-porous species consist almost entirely of earlywood, whereas latewood is confined to a very narrow terminal zone mostly made of ground tissue [15]. Early earlywood (EE) and late earlywood (LE) vessels are respectively positioned within the first and second half of the earlywood (Figure 1). Anatomical measurements of the cells were performed from images of the microsections captured at 50× magnification with a digital camera mounted on a light microscope. The software WinCELL Pro V 2004a [18] was used to measure the number of vessels and, for each of them, the lumen area as well as the radial and tangential lumen diameter. The vessels were then converted into circular conduits following the equation d = [32 (a b)^3^/(a^2^+b^2^)]^1/4^, where d is the circular lumen diameter, and a and b the radial and tangential lumen diameter, respectively [19]. The position (coordinates) of vessels permitted to group them according to two radial classes (EE and LE) and to five tangential classes (5×2 mm out of the 10 mm wide segment examined).

### Statistical Comparisons between the two Study Rings

One-way ANOVA was used to compare vessel lumen size between the control ring and the injury ring, and was performed for each tree using the non-averaged lumen area/diameter of all the vessels analyzed. In addition, Wilcoxon-Mann-Whitney test was used to determine an adequate sample depth, i.e. an adequate number of cells, such that significant (*P*<0.05, *P*<0.01, and *P*<0.001) differences in lumen area were verified between normal vessels and regenerative vessels. Sample depth values were considered adequate when vessel sampling showed 95 to 100% of significant *P*-values (optimum criterion) and where an additional increase of cells bearing the same information did not further improve injury detection (representative criterion). *P*-value percentage calculations were run with the software R 2.14.0 [20] and were based on 100 sampling iterations simultaneously conducted in both rings, where vessels were randomly extracted with replacement (bootstrapping method) and statistically compared. First, automated vessel extraction was independently executed within the whole earlywood (WE), the early earlywood (EE), and the late earlywood (LE). For each specific portion of the ring and for each tree, percentage of significant *P*-values was plotted as a function of sample depth and the described routine was applied in sequence to evaluate the chance of injury detection when progressively increasing the number of vessels. Second, sample depth along with adequate sample location within the 10 mm wide segment of the injury ring were researched in more details for each tree. Vessels in both study rings were thus assigned to the two radial classes (EE and LE) and to the five tangential classes (5×2 mm) previously mentioned. In each portion of the segment, similar *P*-value percentage calculations were computed on subsets of (a) 10, (b) 20, and (c) 30 vessels.

## Results

**Figure 2:**
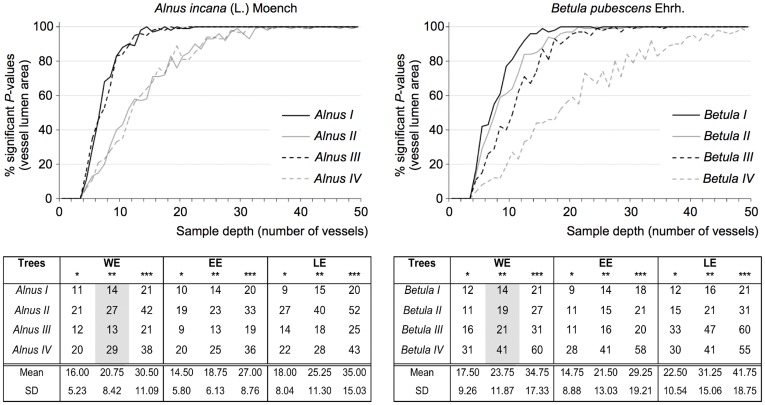
Adequate sample depth for the eight individuals investigated. Sample depth values were considered optimal when vessel sampling showed 95 to 100% of significant *P*-values (Wilcoxon-Mann-Whitney test, * *P*<0.05, ** *P*<0.01, *** *P*<0.001). *P*-value percentage calculations were independently performed within the whole earlywood (WE), the early earlywood (EE), and the late earlywood (LE). Graphs show percentage of significant *P*-values as a function of sample depth for each individual when considering WE and *P*<0.01 (cf. grey column in tables).

Both *A. incana* and *B. pubescens* showed a very highly significant (Table 1, ANOVA test, *P*<0.001) reduction in vessel lumen size between the control ring and the injury ring, ranging from 42 to 64% for lumen area and from 20 to 39% for lumen diameter. Wounding also induced a diminution in the number of vessels (up to 68%). The minimal number of cells necessary for injury detection was very strongly negatively correlated with the magnitude of vessel narrowing (*r* = −0.91, WE, *P*<0.001). For instance, cambial injury caused average vessel lumen area (AVLA) to decrease by 61% in *Betula I* (Table 1), which necessitated the measurement of 9 vessels to identify the disturbance in the injury ring (Figure 2, EE, *P*<0.05). *Betula IV*, in comparison, displayed a lower AVLA decrease (42%) and thus required a larger sample depth (28 vessels).

**Table 1:** One-way ANOVA results for the eight individuals investigated.

Trees	Variables	Control ring (mean ± SD)	Injury ring (mean ± SD)	*P*-value	Change (%)
*Alnus I*	AVLA (µm^2^)	939.70±436.13	381.75±143.31	<0.001	−59
	AVLD (µm)	69.11±17.22	43.76±8.74	<0.001	−37
	VN	1074	486		−55
*Alnus II*	AVLA (µm^2^)	1323.44±669.43	684.80±332.87	<0.001	−48
	AVLD (µm)	80.11±21.76	59.06±14.98	<0.001	−26
	VN	1774	767		−57
*Alnus III*	AVLA (µm^2^)	1130.37±533.41	472.45±191.52	<0.001	−58
	AVLD (µm)	74.78±18.93	48.70±10.39	<0.001	−35
	VN	1825	583		−68
*Alnus IV*	AVLA (µm^2^)	1314.29±656.48	688.50±317.58	<0.001	−48
	AVLD (µm)	83.56±22.92	59.85±14.61	<0.001	−28
	VN	2390	1233		−48
*Betula I*	AVLA (µm^2^)	2096.60±998.67	827.61±424.07	<0.001	−61
	AVLD (µm)	103.93±26.73	63.65±16.56	<0.001	−39
	VN	1037	590		−43
*Betula II*	AVLA (µm^2^)	2050.40±1147.31	737.25±298.69	<0.001	−64
	AVLD (µm)	101.05±30.25	61.52±13.45	<0.001	−39
	VN	1140	404		−65
*Betula III*	AVLA (µm^2^)	1696.99±905.18	753.63±410.48	<0.001	−56
	AVLD (µm)	91.26±25.20	62.33±17.76	<0.001	−32
	VN	1098	421		−62
*Betula IV*	AVLA (µm^2^)	1199.22±653.68	694.99±346.04	<0.001	−42
	AVLD (µm)	76.21±21.60	61.11±16.08	<0.001	−20
	VN	836	580		−31

The average vessel lumen area (AVLA), the average vessel lumen diameter (AVLD), and the number of vessels (VN) were calculated in the injury ring built during the growing season following wounding and in the control ring laid down the previous year. Changes (%) in vessel lumen size between the two rings were tested for significance with one-way ANOVA, performed for each tree using the non-averaged lumen area/diameter of all the vessels analyzed.

Increasing the number of vessels to 20 substantially raised the percentage of significant *P*-values, as illustrated in the graphs of Figure 2 (WE, *P*<0.01). Wound-induced reduction in vessel lumen size was more easily identifiable in EE than in LE where more cells (47% on average, *P*<0.05) needed to be analyzed to reach 95 to 100% of significant *P*-values (Figure 2). It is however clear that, no matter the extent of earlywood, the higher the level of significance the larger the sample depth required. For example, sample depth values averaged over the four individuals indicated that injury detection in *A. incana* could be accomplished through the measurement of 31 vessels within WE, 27 within EE, and 35 within LE (Figure 2, *P*<0.001). Slightly larger values were reported for *B. pubescens* (35, 29 and 42, respectively) due to *Betula IV*, characterized by a weaker vessel narrowing and therefore larger sample depth values.

**Figure 3:**
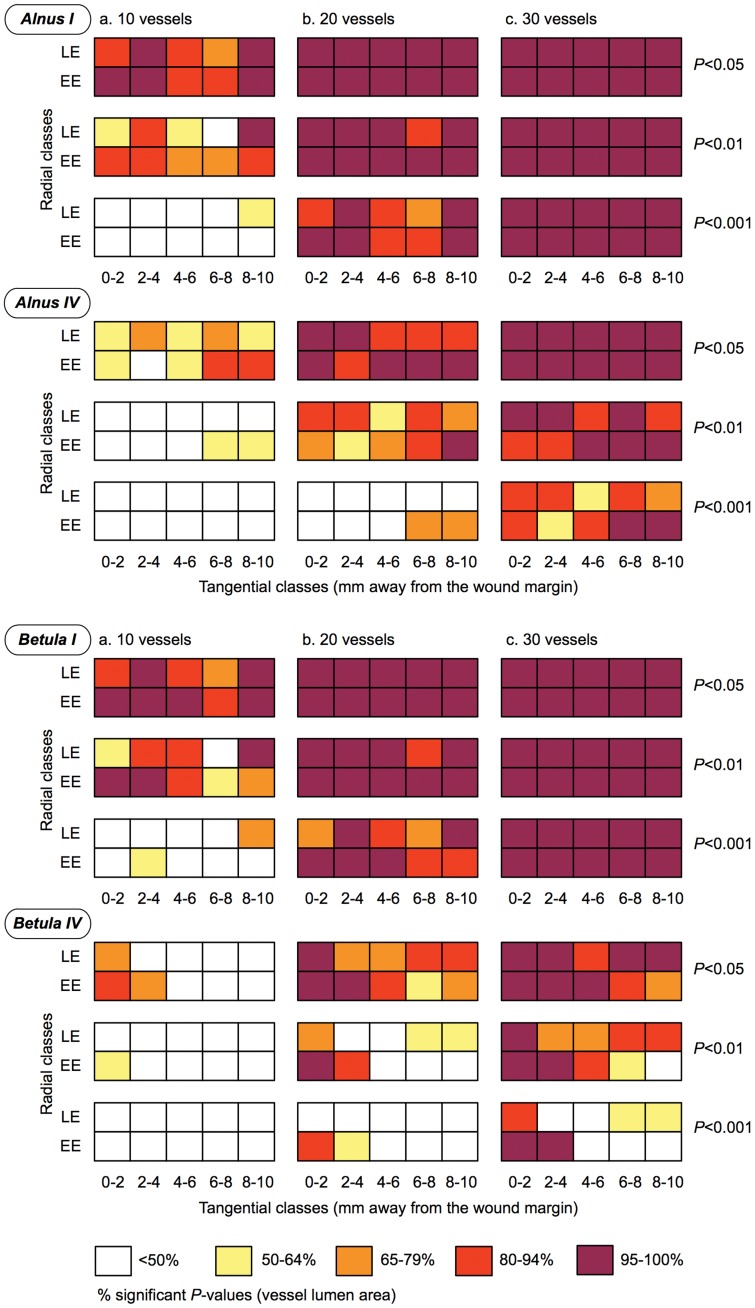
Adequate sample depth and adequate sample location for two representative individuals per species. Sample depth values were considered optimal when vessel sampling showed 95 to 100% of significant *P*-values (Wilcoxon-Mann-Whitney test, *P*<0.05, *P*<0.01, *P*<0.001). The 10 mm wide segment investigated in both study rings was divided into 10 portions according to two radial classes (EE, early earlywood and LE, late earlywood) and to five tangential classes (5×2 mm). In each portion of the segment, *P*-value percentage calculations were computed on subsets of (a) 10, (b) 20, and (c) 30 vessels.

Furthermore, the matrices presented in Figure 3 allowed three subsets of vessels (10, 20, and 30) and three levels of significance (*P*<0.05, *P*<0.01, and *P*<0.001) to be tested in order to delineate general tendencies. Measuring 10 vessels in each earlywood compartment resulted in <50% of significant *P*-values, particularly in individuals with a lower AVLA decrease, such as *Alnus IV* and *Betula IV* (Table 1, Figure 3). Outcomes were unsatisfactory for *P*<0.001 and strongly individual-dependent for *P*<0.05. Doubling the number of measurements from 10 to 20 produced results that were still impeded by individual variability for *P*<0.001 and *P*<0.01. However, for *P*<0.05, analyzing 20 vessels proved to be reliable in each portion of the 10 mm wide segment, except in *Betula IV*. This individual drew attention to the fact that a lower AVLA decrease (42%, Table 1) made cambial injury only detectable close to the wound margin (within the first 2 mm, Figure 3). When analysis was based on 30 vessels, outcomes were again strongly individual-dependent for *P*<0.001, but were satisfactory for *P*<0.01 and excellent for *P*<0.05. In both latter cases, it is interesting to note that wound-induced reduction in vessel lumen size was identified in the two radial classes (EE and LE) and in the five tangential classes (5×2 mm).

## Discussion

The results of this study for *A. incana* and *B. pubescens* corroborate the formation of narrower vessels after wounding [21]–[24]. Moreover, the magnitude of vessel narrowing was observed to be individual-dependent rather than species-dependent. The occurrence of vessel anomalies as a reaction to mechanical disturbance emphasize the crucial importance of vessels in tree metabolism as they are thought to be involved in a trade-off triangle comprised of three competing functions – water transport, mechanical support, and resistance to embolism [25]. More precisely, environment-driven changes in wood anatomical features represent adaptative structural solutions adopted by the tree in order to achieve an optimal balance among multiple competing needs including water transport, water and carbon storage, mechanical support, resistance to embolism and resistance to decay [26]–[27]. Wound-induced vessel anomalies have been attributed to changes in auxin and ethylene concentrations [28]–[30], and have only recently been employed to date scars inflicted on trees by mass-movement processes [13], [16], [27], [31].

### Adequate Sample Depth for Injury Detection

Our findings for *A. incana* and *B. pubescens* show that a minimum of 20 vessels is required to attest to cambial injury through vessel lumen size reduction, which is in agreement with St. George et al. [3] who argued that 20 vessels were enough to furnish a good estimate of mean vessel size for *Quercus macrocarpa* Michx. However, more detailed observations from the present study suggest that 20 cells would only be sufficient for *P*<0.05 and for those scarred trees that developed a rather strong vessel narrowing. The minimal number of cells necessary for injury detection is indeed very strongly negatively correlated with the magnitude of vessel narrowing. We therefore recommend measuring at least 30 vessels in each ring so as to produce results that are less impeded by individual variability. According to the fact that the higher the level of significance the larger the sample depth required, we estimate that 30–40 vessels would be appropriate for *P*<0.05 and *P*<0.01, whereas 40–50 vessels would be a more sensible choice for *P*<0.001. It was also found that, in comparison to EE, more cells need to be analyzed within LE to obtain an adequate sample, certainly due to the fact that LE conduits are usually narrower [15].

### Adequate Sample Location for Injury Detection

EE and LE vessels can be equally used for that purpose, which indicates that in the case of scars inflicted on trees prior to the growing season reduction in vessel lumen size do not substantially diminish throughout earlywood formation in *A. incana* and *B.* *pubescens*. While we report no radial fading of vessel narrowing in the first ring built after wounding, we would however expect injury detection in the second ring onwards to be challenged by a prompt recovery in vessel lumen size, as demonstrated for certain diffuse- and ring-porous species [16], [27]. It is in fact well acknowledged that wound-induced anatomical and physiological reactions decline with increasing distance from the injured area [32]–[34], presumably in either direction – longitudinal, radial, and tangential. In regard to the tangential extent of vessel narrowing though, results seem to vary between diffuse- and ring-porous species. Arbellay et al. [27] determined that in young *Fraxinus excelsior* L. (ATA 17.11±4.46 yr, ASC 8.03±2.32 cm) earlywood vessels produced the first year after wounding were narrower only within approximately 4.5 mm from the wound margin. By contrast, we established that in *A. incana* and *B. pubescens* (ATA 29.50±12.93 yr, ASC 21.36±8.42 cm) narrower conduits were formed within 10 mm from the wound margin. It appears that in the tangential direction wound-induced reduction in vessel lumen size does not fade as radically in diffuse-porous species as in ring-porous species. Moreover, they have been shown to persist over the entire stem circumference one year after the scarring event in *A. incana* and *Betula pendula* Roth [13]. We therefore argue that injury detection is not restricted to any particular radial or tangential sample location, although the early earlywood vessels located closest to the injury should still be preferred given the fact that scarred trees may develop a weak vessel narrowing.

In conclusion, despite the inherent individual variability, this study furnishes important guidelines for retrospective injury detection in diffuse-porous species. Extensive calculations helped refine the number and the intra-ring location of cells required to attest to cambial injury. The results of this study also provide a theoretical basis for future related research on hydraulic parameters in wounded trees.
